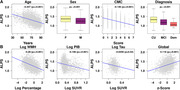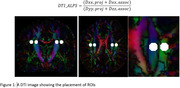# Evaluating DTI‐ALPS, an imaging surrogate for glymphatic clearance, in a large population‐based sample

**DOI:** 10.1002/alz.087185

**Published:** 2025-01-09

**Authors:** Siddhartha Satpathi, Robert I. Reid, Scott A. Przybelski, Sheelakumari Raghavan, Jeffrey L. Gunter, David S. Knopman, Ronald C. Petersen, Petrice M Cogswell, Clifford R. Jack, Jonathan Graff Radford, Prashanthi Vemuri

**Affiliations:** ^1^ Mayo Clinic, Rochester, MN USA; ^2^ Department of Quantitative Health Sciences, Mayo Clinic, Rochester, MN USA; ^3^ Department of Radiology, Mayo Clinic, Rochester, MN USA; ^4^ Department of Neurology, Mayo Clinic, Rochester, MN USA

## Abstract

**Background:**

Diffusion tensor imaging along perivascular spaces index (DTI‐ALPS), which measures diffusivity increases in the perivascular spaces along the medullary veins, is being increasingly utilized as a surrogate marker of glymphatic clearance (Taoka et. al. Jpn J Radio 2017). Lower DTI‐ALPS index means higher measured diffusivity from the perivascular space, and so it is expected to decrease with age. However, correlation with aging and dementia markers have not been systematically evaluated. The goal of this work was to automatically estimate DTI‐ALPS in a large sample and evaluate the variance explained by various demographic and imaging markers.

**Methods:**

We identified 2739 participants aged 30+ years (mean 69.6, 48% female, 88% CU) in the population‐based Mayo Clinic Study of Aging with diffusion MRI. We estimated DTI‐ALPS through automated placement of regions as published previously (Liu et al. AAIC 2023) and shown in Figure 1. We examined the associations of DTI‐ALPS with demographics (age, sex), vascular risk (cardiovascular and metabolic condition score), clinical data (diagnosis, global cognition) and imaging markers (white matter hyperintensity (WMH), global amyloid load from PIB‐PET, and the temporal meta‐ROI Tau‐PET SUVR) using partial Pearson correlations and ANCOVA with adjustments for age and sex.

**Results:**

DTI‐ALPS was negatively correlated with age, higher in females, declined with vascular risk, and lower in MCI/dementia groups (Figure 2). Higher WMH and higher amyloid were associated with lower DTI‐ALPS whereas Tau‐PET SUVR was not associated with DTI‐ALPS.

**Conclusions:**

DTI‐ALPS can be automatically computed in large samples. Vascular risk and WMH had stronger associations with the computed DTI‐ALPS compared to AD biomarkers (amyloid and tau) suggesting that the surrogate was more likely linked to vascular injury.